# The Importance of Maintaining Protected Zone Status against *Bemisia tabaci*

**DOI:** 10.3390/insects6020432

**Published:** 2015-05-11

**Authors:** Andrew G. S. Cuthbertson, Irene Vänninen

**Affiliations:** 1 Fera, Sand Hutton, York YO41 1LZ, UK; 2 Natural Resources Institute Finland, Tietotie 2C, Jokioinen FI-31600, Finland; E-Mail: irene.vanninen@luke.fi

**Keywords:** *Bemisia tabaci*, eradication, protected zone

## Abstract

The sweetpotato whitefly, *Bemisia tabaci* (Gennadius) (Hemiptera: Aleyrodidae) is a major pest of economically important crops worldwide. Both the United Kingdom (UK) and Finland hold Protected Zone status against this invasive pest. As a result *B. tabaci* entering these countries on plants and plant produce is subjected to a policy of eradication. The impact of *B. tabaci* entering, and becoming established, is that it is an effective vector of many plant viruses that are not currently found in the protected zones. The Mediterranean species is the most commonly intercepted species of *B. tabaci* entering both the UK and Finland. The implications of maintaining Protected Zone status are discussed.

## 1. Introduction

The sweetpotato whitefly, *Bemisia tabaci* (Gennadius) (Hemiptera: Aleyrodidae) is a major pest of economically important crops worldwide [1]. *Bemisia tabaci* damages crops by feeding on phloem sap and the large amounts of sticky honeydew produced can lower the rate of leaf photosynthesis. This whitefly is also a vector of many plant viruses [2,3,4].

The pest status of *B. tabaci* insects is complicated by the recognition of 11 well-defined genetic groups and at least 34 morphocryptic species, which are morphologically identical but distinguishable at the molecular level [5,6,7,8]. Formerly, the term biotype was used to define and discriminate *B. tabaci* populations with very different biological characteristics, including invasiveness, insecticide resistance profile, vector competence, and host ranges [9]. It is the former B biotype (now known as Middle East-Asia Minor 1 (MEAM1) species) and Q biotype (now known as Mediterranean species) that are the most invasive and damaging *B. tabaci* species around the world, presenting the greatest threat to glasshouse crops [10,11]. The damaging MEAM1 is an aggressive colonizer and it is an effective vector of viruses, whereas the Mediterranean species characteristically shows strong resistance to novel insecticides [10,12,13].

## 2. Economic Importance of Protected Zone Status

*Bemisia tabaci* is listed in the European Union (EU) Plant Health Directive 2000/29/EC under Annex 1AI (non-European populations) as a harmful organism, whose introduction from non-EU countries into, and spread within, all EU member states shall be banned [14]. The United Kingdom (UK) and Finland are currently two member states (along with Sweden, Republic of Ireland and parts of Portugal) that remain free of all populations of *B. tabaci* and maintain Protected Zone (PZ) status against this pest [14,15,16]. The establishment of *B. tabaci* within a Protected Zone area, for example the UK, poses a threat to the horticultural industry, in particular to the tomato industry. MEAM1, in particular, can transfer and infect tomatoes with both Tomato Yellow Leaf Curl Virus (TYLCV) and Tomato Yellow Leaf Curl Sardinia Virus (TYLCSV) and, depending on the timing of infection, losses can reach 100% [17]. The only reference to the economic burden of *B. tabaci* and its associated viruses becoming established in the UK is outlined in Morgan and MacLeod [18]. It is estimated that a single producer might reduce their profit margins by around £5500 per 0.1 ha, while the national industry might suffer losses of around £11.5M. Though outdated, these figures highlight the need for suitable pest management strategies to be developed to avoid significant financial loss to the UK tomato industry.

## 3. *Bemisia tabaci* Interceptions

Within Europe, *B. tabaci* is established in the Mediterranean coastal regions and, even with an estimated temperature increase of 2°C, expansion of areas where the pest can establish will still be very distant from the northern European countries with PZ status [19]. The PZ status for Portuguese islands, in which climatic conditions are suitable for *B. tabaci*, is maintained only by exclusion of the pest.

**Figure 1:**
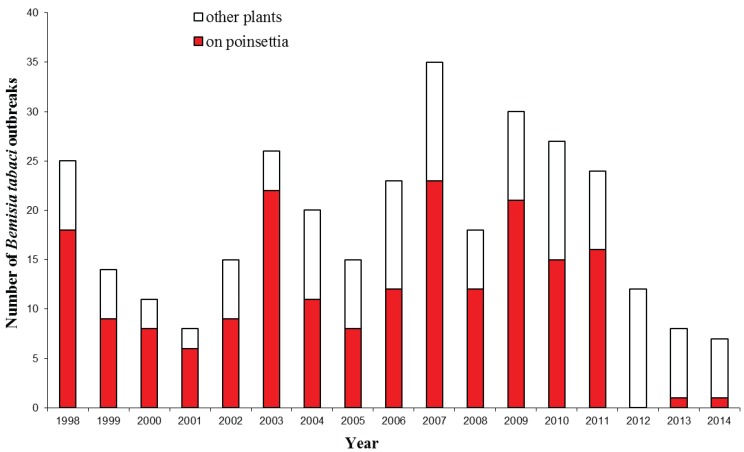
The number of *Bemisia tabaci* outbreaks in England and Wales (1998‒2014) (updated from Cuthbertson [15]).

Within the UK, *B. tabaci* has been intercepted at growing sites on an extremely wide range of hosts at nurseries since 1987 [15]. Interceptions of *B. tabaci* coming into the UK would appear to follow no pattern with numbers of interceptions from source countries varying widely and with the majority of outbreaks being associated with *Euphorbia pulcherrima* (poinsettia) plants (Figure 1) [15,16]. Historically, it has been MEAM1 that has been entering the UK, but recently there has been a shift to Mediterranean species [7,20]. Mediterranean species is widely considered to evolve stable resistance to neonicotinoid insecticides more rapidly when compared to MEAM1 species [21]. As a result, Mediterranean species neonicotinoid resistance is becoming increasingly widespread and problematic, with numerous cases being reported world-wide [22,23].

Both the Mediterranean and MEAM1 species are also regularly intercepted and, as a result, undergo a process of eradication in Finland [24]. The majority of outbreaks in production places, having numbered 16‒83 per year since 2009, have been on poinsettia originating from a number of countries in the EU [25]. During recent years, however, *Bemisia* has also increasingly entered on imported bedding and pot plants, particularly on *Mandevilla*, in the spring-time [26]. This development may, in principle, increase the potential of the species spreading between infested production places during the summer time before eradication is complete, or when plants with non-detected *Bemisia* are planted outdoors. The Mediterranean species, due to its tendency of resistance development and, among other things, better development rates at both high and low temperatures [27], is estimated to pose a somewhat bigger threat to Finnish plant production than MEAM1 [28].

## 4. Impact of *Bemisia tabaci* Establishment

Impact is predominantly related to diseases caused by *B. tabaci* transmitted viruses on crops and particularly by those begomoviruses causing yellow leaf curl diseases in tomato, which have a severe impact on crop cultivation and yield in all regions where tomato is grown. The synergy between the two highly invasive whitefly species (MEAM1 and Mediterranean) and both TYLCV and TYLCSV, which are highly infectious to tomato (as well as pepper and beans), efficiently transmitted by *B. tabaci* and adaptable to environments where tomato is cultivated, are characteristics of an invasive virus. Most *B. tabaci* transmitted viruses cause high impact in crops and, generally, losses from those virus diseases can be considered as very economically important [17].

Thus far there are no known introductions of *Bemisia*-associated viruses in Finland, and the likelihood of TYLCV/TYLCSV entering and establishing in Finland via the pathway of imported ornamental plants is considered small [14]. This is because ornamental plants imported into the country, with the exception of *Lisianthus* [29] are either not hosts, or are not good hosts, for the virus [14]. Furthermore, vegetable plants for planting do not form an important pathway of introduction for the insect or viruses, at least not for the moment, since professional Finnish vegetable producers only use domestic plants for planting. Such functional isolation from the main introduction pathways, coupled with unsuitable outdoor conditions for permanent establishment of the insect itself, scarcity of TYLCV/TYLCSV natural host plants outdoors that could serve as a reservoir for the virus, and the presumably low back-transmission of the virus from non-solanaceous hosts to tomato [30], are all bonus factors that, when coming together, help preserve the PZ area free of *Bemisia* and TYLCV/TYLCSV.

There are three major knowledge gaps with respect to the impact of *Bemisia*-transmitted plant viruses in the PZs. Firstly, it is not known what proportion of the intercepted insects are viruliferous, and how often virus-infested but symptomless plants enter the country together with the vector. It is known that viruliferous insects do sometimes arrive into PZ areas as TYLCSV has been recently detected in *B. tabaci* entering the UK [20]. Secondly, it is not known how effectively the vector can acquire and transmit TYLCV/TYLSCV from infested but symptomless plants such as *E. pulcherrima* that are regularly imported to the PZ countries and are often infested with *B. tabaci*. The vector can acquire TYLCV/TYLCSV from some symptomless non-solanaceous host plants [31], whereas from others acquisition rates are very low due to low titres of virus in the symptomless plants [30]. Finally, the likelihood of *B. tabaci*, either with or without plant viruses, moving from ornamentals to vegetable production in the PZ countries is not known. For viruses entering with *B. tabaci* on ornamentals, there is a risk of outbreaks when tomato and ornamentals are grown in the same environment at the same time. Such virus outbreaks can be eliminated if detected in time, as exemplified by an eradication campaign conducted in The Netherlands in 2007 [32], specifically when the vector is not present. TYLCV has been shown to arrive also in imported fruits [33], and the virus can be acquired and transmitted from tomato fruit by the vector [34]. However, the transfer of the virus from such fruits to vegetable production is impossible unless the vector is also present, gets access to the fruits and then re-enters vegetable glasshouses.

The likelihood of *B. tabaci* moving from ornamentals to vegetable production needs to be understood better to be able to estimate impacts of *B. tabaci* establishing in PZ countries. Imported ornamentals were most likely the initial access route for the glasshouse whitefly *Trialeurodes vaporariorum* to Finnish vegetable production; currently this pest is causing the most problems in vegetable production owing to changes in production strategies. Heikkilä and Vänninen [35] used stochastic spatial simulation modeling for analyzing what would happen under different scenarios of *B. tabaci* spreading from ornamentals to vegetable production. The likelihood of the move depends on issues such as the geographical proximity of ornamental and vegetable production sites, the extent to which the pest can be controlled in ornamental production, and the logistic networks of traded ornamentals within the country. These factors are coupled with outdoor conditions that impact pest mortality to a higher or lesser extent should the pest attempt spreading through the outdoor environment. The results are likely to differ by PZ country since conditions influencing the spread of the pest vary.

The establishment potential and impacts of the pest itself, in the absence of plant viruses, should be estimated taking specific characteristics of glasshouse production in each PZ into account. A characteristic feature of Finnish glasshouse horticulture is year-round production using artificial lighting. In these conditions, *T. vaporariorum* can be used as a proxy for exploring the establishment, spread and impacts of *B. tabaci*. In production clusters particularly, the advent of year-round production from the 1990s onwards has resulted in region wide management difficulties of the glasshouse whitefly in both the new and the old (seasonal) production forms. The growers routinely apply preventive control against the glasshouse whitefly in both tomato and cucumber, since practically all crops get infested at some point of the cropping cycle [36]. Year-round crops would provide good conditions for reproduction and survival of *B. tabaci* in terms of temperature [37] and daylength of 16‒20 hours [38,39]. Uninterrupted cropping cycles would enable the production of 8‒10 generations per year. It is likely that the year-round production, with time, would enable the establishment and uninterrupted persistence of *B. tabaci* in Finnish vegetable production in the same way as *T. vaporariorum* now persists and spreads there, especially in production clusters [40,41].

## 5. Maintaining Protected Zone Status

If PZ status was to be lifted for PZ countries, restrictions from Annex IV, Part A, Section I banning introduction and spread of non-European *B. tabaci* populations would still apply. Revoking the status would place PZ countries in a similar position to other northern countries where *B. tabaci* cannot establish, with systematic inspections limited to material imported from outside the EU. For host material produced within the EU, there would be no routine response to outbreaks or pre-emptive measures including statutory inspections. A free flow of EU traded commodities would likely increase occurrences and outbreaks of *B. tabaci* and with that the likelihood that viruses are transmitted to significant crops, tomato and cucurbits, causing high impact and severe crop losses. Maintaining the PZ status with the current Annex 1AI status would still support the zero tolerance approach towards *B. tabaci*, regardless of its origin with the benefit of a significantly reduced risk of entry of *B. tabaci* and the viruses it transmits. A UK Plant Health consultation on the future of PZ status in the UK [42], had an overwhelming response to maintain the status quo.

According to Defra [42], calculated cost benefit ratios of several impact scenarios were produced for protected salad crops (cucumber and tomatoes). The ratios ranged from 1:1.2 to 1:30 depending on the scenario and crop; these results therefore would support maintenance of the PZ. The analysis in the Defra [42] report concludes that although the total annual costs (to both industry and government) of eradicating *B. tabaci* are large, they are considerably less than the potential benefits obtained by excluding the pest. The potential impact of the pest (and vectored diseases) in terms of crop losses in salad crops potentially being much more important than the effect of the pest in terms of increasing the costs associated with controlling it. The loss of PZ would mean that growers of ornamental crops would have to control (but not eradicate) both *B. tabaci* and *T. vaporariorum*; however, salad growers could be confronted for the first time with the problem of controlling *B. tabaci* as well as *T. vaporariorum* whereas currently they have only had to deal with *T. vaporariorum*. Studies suggest that in tomato *B. tabaci* can replace *T. vaporariorum* due to the former’s superior competitive abilities [43]. Having to deal with two or more pest species that are dynamically establishing their competitive roles adds to the complexity of pest management.

In Finland, the debate about PZ status has been accentuated by a new plant health law according to which eradication costs of *B. tabaci* are no longer compensated for by government funds. Even so, the status quo was maintained due to risks associated with the *Bemisia*-vectored plant viruses and the specificities of Finnish vegetable production with highly conducive conditions for the pest’s reproduction. A cost-benefit analysis (CBA) of the PZ for a number of scenarios was conducted by Heikkilä [44]. The three scenarios for maintaining the PZ assumed that the annual number of infested ornamental glasshouses either remains at 50 per year, or doubles or triples by 2038. The two scenarios for abolishing the PZ assumed either that *Bemisia* spreads in six years to all poinsettia and in 30 years to about one sixth of the total cucumber and tomato area, or alternatively that the spread on tomato is faster than this. Accounting for uncertainty regarding the costs of control, the benefit:cost ratio was estimated to be 0.52‒2.63. This means that it could not be established with certainty whether to abolish or to maintain the PZ. Given the baseline parameter values, however, five of the six scenario comparisons favoured maintaining the PZ. The CBA also concluded that if *Bemisia* would spread to tomato or cucumber production and cause even moderate crop losses, maintaining the PZ would always be profitable. If, on the other hand, the risk of *Bemisia* spreading to vegetable production was small and the costs of control on flower farms remained moderate, then giving up the PZ could be an option. The CBA did not include *Bemisia*-associated viruses in the scenarios, but only the insect itself as a pest.

The Mediterranean species of the *B. tabaci* species complex predominates in specimens entering both the UK and Finland. Therefore, control and eradication programmes need to be able to deal with this species [45]. Most importantly, the propensity for Mediterranean species to develop strong insecticide resistance very clearly suggests that the control options available in PZ countries may not always be sufficient to eradicate the pest, particularly in outbreak situations in ornamental nurseries [46]. Furthermore, *B. tabaci* species other than MEAM1 or Mediterranean are now also becoming more common on imported plant material [47]. Determining their status in the future is also of importance in order to fully understand the consequences of their potential outbreak and establishment. As a result, monitoring for *Bemisia* cryptic species and resistance status, as well as the presence of plant viruses in the intercepted insects [20], are important facets in ensuring that PZ glasshouse horticulture remains free from this highly damaging invasive pest in the future.

## 6. Conclusions

*Bemisia tabaci* is a worldwide pest and major virus vector. Originally known as a pest of sub-tropical crops, the species is now widely distributed under glass in temperate areas including most of Europe and on outdoor crops in southern Europe. Currently, it is not established in the UK or Finland. However, it could establish in protected environments, where it has the potential to be a major pest, particularly of glasshouse salad crops such as tomato and cucumber, given its potential to spread damaging viruses. The co-existence of year-round and seasonal vegetable production may specifically increase the establishment potential of the pest if it was to move from ornamental production to vegetable crops. The maintenance of protected zone status offers countries like the UK and Finland better control over their respective borders in regards to plant imports and in terms of protecting against introductions on EU traded material. Keeping the respective countries free from highly damaging plant viruses is vital to maintaining a healthy protected horticultural industry.
